# Differential Preferences in Breast Aesthetics by Self-identified Racial Demographics Assessed on a National Survey

**DOI:** 10.1093/asjof/ojad027.010

**Published:** 2023-04-14

**Authors:** Carter Boyd, Jonathan Bekisz, Kshipra Hemal, Mihye Choi, Nolan Karp

**Affiliations:** NYU Langone Health, New York, NY; NYU Langone Health, New York, NY; NYU Langone Health, New York, NY; NYU Langone Health, New York, NY; NYU Langone Health, New York, NY

## Abstract

**Goals/Purpose:**

Breast aesthetics are a highly discussed topic within plastic surgery. However, there is a paucity of literature examining how preferences differ amongst various demographic groups. The objective of this study was to assess how perceptions of the ideal breast differ between respondents stratified by self-identified race.

**Methods/Technique:**

A cohort of 25 patients from the senior surgeons’ practices presenting for aesthetic or reconstructive breast surgery was assembled. This cohort was selected with the aim of choosing a sample that maximized diversity on the basis of breast size and shape, skin tone, and nipple-areola complex size and position. Pre-operative anteroposterior photographs from these patients were distributed via Qualtrics (Seattle, WA) to a large sample designed to be representative of the demographics of the United States. Survey questions aimed to measure respondents’ impressions of “breast attractiveness.” Respondents were asked to rate breasts on a Likert scale of 1 to 5, with 1 being not at all attractive and 5 being most attractive. Survey responses were analyzed for differences in breast aesthetic preferences by race using ANOVA and t-tests where appropriate.

**Results/Complications:**

A total of 1,021 survey responses were collected. The majority of survey respondents identified as White or Caucasian (73%). A total of 18% of individuals self-identified as Black or African-American, while 5% identified as Asian. The remaining 3% of respondents identified as Other, Native American, or selected multiple categories and groups were too small for cross-group comparison and were thus excluded. On average, individuals in the White or Caucasian group rated any given pair of breasts 8% more attractive than Black or African-American individuals and 14% more attractive than Asian individuals. ANOVA testing revealed significant differences amongst groups (p=0.014). Subgroup comparison revealed a significant difference between ratings of breast attractiveness between White or Caucasian individuals and Asian respondents (2.61 vs 2.25; p=0.002). Comparisons between White or Caucasian respondents and Black or African-American individuals were not significant (2.61 vs 2.40; p=0.06) as was the analysis between Black or African-American respondents and Asian individuals (2.40 vs 2.25; p=0.08). While these intergroup comparisons did not reach significance in the current sample, perhaps with a larger sample size a difference between these groups might be elucidated. Despite these racial discrepancies in breast attractiveness, regression analysis revealed that the ratings amongst each of the groups were highly correlated with one another: White or Caucasian and Black and African-American (R=0.68; R^2^=0.46; p<0.001), Black or African-American and Asian (R=0.72; R^2^=0.52; p<0.001), and White or Caucasian and Asian (R=0.86; R^2^=0.73; p<0.001).

**Conclusion:**

In a sample representative of the United States, a difference in breast aesthetic appraisal was observed by race. On average, White or Caucasian respondents rated a given pair of breasts as more aesthetically pleasing than Black or African-American and Asian counterparts. While this difference was observed, it is important to note that ratings amongst racial groups were highly correlated with one another across all photographs presented. In other words, while Black or African-American and Asians rated breasts lower than their White or Caucasian counterparts, relative attractiveness for the breasts amongst groups paralleled one another. While self-identified racial groups rated the breasts similar relative to one another, there were distinct differences in numerical ratings that may be reflective of broader, more generalized sentiments and preferences that were not able to be captured by our survey instrument. These findings merit further investigation to understand these trends and observations. Doing so will enable plastic surgeons to have a better understanding of patient preferences and expectations when seeking reconstructive or aesthetic breast surgery and thereby improve patient satisfaction.

